# Differential Expression of miRNAs and Their Predicted Target Pathways in Cochlear Nucleus Following Chronic Noise Exposure in Rats

**DOI:** 10.3390/cells11152266

**Published:** 2022-07-22

**Authors:** Chang Ho Lee, Jiwon Jeon, So Min Lee, So Young Kim

**Affiliations:** Department of Otorhinolaryngology-Head & Neck Surgery, College of Medicine, CHA University, Seongnam 13496, Korea; hearwell@gmail.com (C.H.L.); tw7682@nate.com (J.J.); lws6812@naver.com (S.M.L.)

**Keywords:** microRNAs, noise, hearing loss, cochlear nucleus, AKT3, SIRT1

## Abstract

Several recent preclinical studies have reported that dynamic changes in miRNA expression contribute to hearing function. This study aims to investigate miRNA expression changes in the cochlear nuclei (CN) of rats following chronic noise exposure. Eight-week-old rats (*n* = 14) were exposed to noise for 4 weeks. The control rats (*n* = 14) were raised under identical conditions without noise. Two months after noise exposure, the auditory brainstem response (ABR) was examined, and the cochlea and CN were harvested. In the CN, the expression levels of arc, neurocan, and brevican were measured (*n* = 6 per group). Furthermore, the expression levels of miRNAs and their predicted target genes were measured in the CN (*n* = 8 per group). ABR thresholds were elevated after 4 weeks of noise exposure, which were maintained for 3 months. In CN, the protein expression of arc and brevican was higher in the noise-exposed group than in the control group (0.95 [standard deviation (SD) = 0.53] vs. 3.19 [SD = 1.00], *p* < 0.001 for arc and 1.02 [SD = 0.10] vs. 1.66 [SD = 0.24], *p* < 0.001 for brevican). The noise-exposed rats exhibited lower expression levels of miR-758-5p, miR-15b-5p, miR-212-3p, miR-199a-5p, and miR-134-3p than the control rats (all *p* < 0.001). The AMPK signaling pathway was predicted to be regulated by these miRNAs. The predicted target genes AKT3, SIRT1, and PRKAA1 were highly expressed in noise-exposed rats. In CN of noise-exposed rats, the miRNAs of miR-758-5p, miR-15b-5p, miR-212-3p, miR-199a-5p, and miR-134-3p were reduced and related to AMPK signaling including AKT3 and SIRT1 expression. These modulation of signaling pathways could mediate the increased expression of brevican in the CN of noise-exposed rats.

## 1. Introduction

The cochlear nucleus (CN) is an important auditory brainstem that controls auditory perception and synaptic plasticity following external stimuli such as noise exposure [1]. The evolutionary auditory hindbrain, including the CN, represents therian-specific features of the auditory nervous system, which may contribute to the high-frequency hearing ability (>15–20 kHz) [2]. The restriction of synaptic plasticity and maintenance of high-frequency synaptic relays have been reported to be linked with high density of perineuronal nets (PNs) in the CN [3]. The chondroitin sulfate proteoglycans (CSPGs), such as brevicans and neurocans, are composed of PNs [4]. Changes in the PNs following noise exposure have been reported in the primary auditory cortex [5,6]. However, the changes in PNs or CSPGs have not been explored in the auditory brainstem of noise-induced hearing loss model.

As short non-coding transcripts, miRNAs orchestrate the transcription or translation of genes during development and pathological conditions, including auditory deprivation [7,8]. As miRNAs are abundant in the central nervous system and harbor multiple target genes, they are thought to regulate the neural pathways of neurogenesis and synaptogenesis [9,10]. For instance, miRNAs may suppress the translation of synaptic mRNAs before exposure to external stimuli such as chronic stress, which can induce synaptic plasticity [11].

Several recent preclinical studies have reported that dynamic changes in miRNA expression contribute to hearing function [7,8,12,13]. miRNAs are presumed to regulate the development of hearing function [7]. Among the 12 abundant miRNAs in the cochlea, 9 show dynamic changes during developmental periods and 11 are differentially expressed in the CN and superior olivary complex of the auditory hind brain [7]. Noise exposure has been suggested to alter miRNA expression in the CN [8]. Even as short as 2 h of noise exposure could induce changes in miRNA expression in the CN, although hearing thresholds were recovered [8]. However, changes in miRNA expression following chronic noise exposure have not been investigated.

Previous studies demonstrated the changes of CSPGs including brevican in auditory nervous system following noise-induced hearing loss [5,6]. The mediating role of miRNAs were suggested for the changes of CSPGs in visual cortex [14]. Therefore, we hypothesized that chronic noise exposure might induce changes in the expression of miRNAs that mediate synaptic plasticity, such as change of CSPGs, in the CN. To test this hypothesis, adult rats were exposed to chronic noise, and miRNA levels were examined. The target genes and signaling pathways for the dysregulated miRNAs were predicted, and the expression levels of CSPGs, neurocan, and brevican were estimated in the CN of noise-exposed rats.

## 2. Results

**Figure 1:**
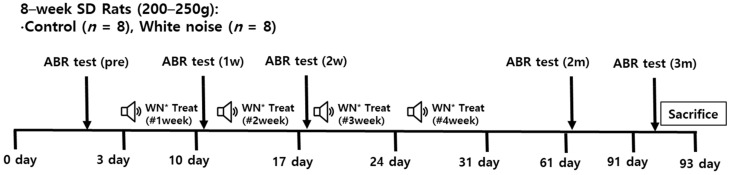
Design of the present study (*n* = 8 per group). Noise was experienced by the rats for 4 weeks, and hearing levels were measured via auditory brainstem response (ABR) tests. The qRT-PCR of miRNA (*n* = 8), the predicted target genes (*n* = 8 per group), and Western blotting (*n* = 6) were conducted using cochlear nuclei of the noise-exposed and control rats. * White noise (115 dB SPL) 3 h/day 3 times a week.

The noise was periodically exposed for 4 weeks in the noise-exposed rats (Figure 1). The hearing levels were measured at 1 week, 2 weeks, 2 months, and 3 months after the noise exposure.

**Figure 2:**
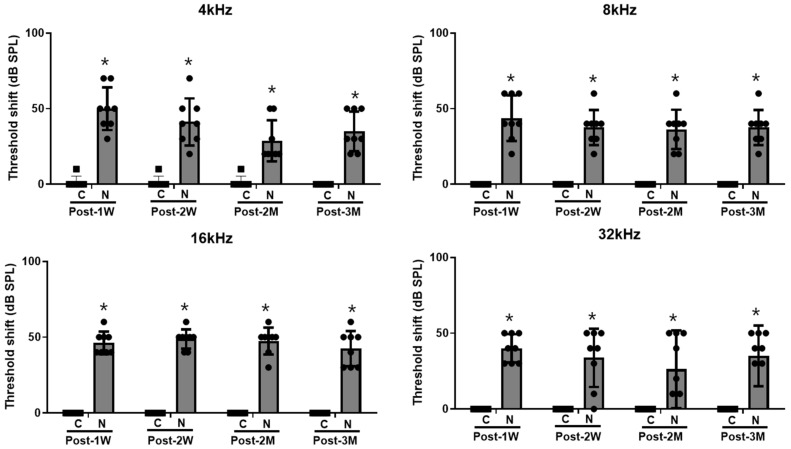
Hearing threshold shifts after noise exposure (*n* = 8 per group). The noise exposed rats demonstrated hearing threshold shifts at 1 week, 2 weeks, 2 months, and 3 months post noise exposure at 4, 8, 16, and 32 kHz. (* *p* < 0.05; unpaired *t*-test between the control and noise-exposed groups; C: control rats; N: noise rats).

The threshold shifts were maintained in the noise-exposure group at 1 week, 2 weeks, 2 months, and 3 months after the noise exposure (all *p* < 0.05) (Figure 2).

**Figure 3:**
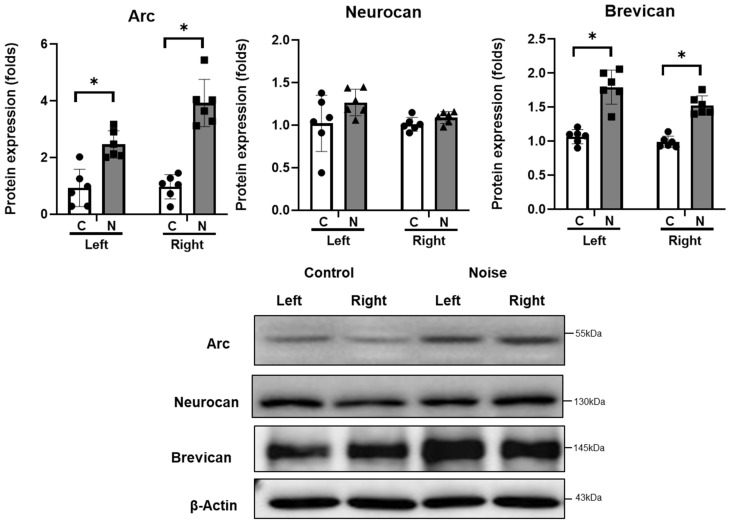
Arc, neurocan, and brevican expression in the cochlear nuclei after noise exposure (*n* = 6 per group). Arc and brevican expression was increased in the noise-exposed group (* *p* < 0.05; unpaired *t*-test between control and noise-exposed rats; C: control rats; N: noise rats).

The protein expression levels of arc were higher in the CN of noise-exposed rats than in those of the control rats (0.95 [SD = 0.53] vs. 3.19 [SD = 1.00], *p* < 0.001) (Figure 3). Brevican was also expressed at higher levels in the CN of noise-exposed rats than in those of the control rats (1.02 [SD = 0.10] vs. 1.66 [SD = 0.24], *p* < 0.001) (Figure 3).

**Figure 4:**
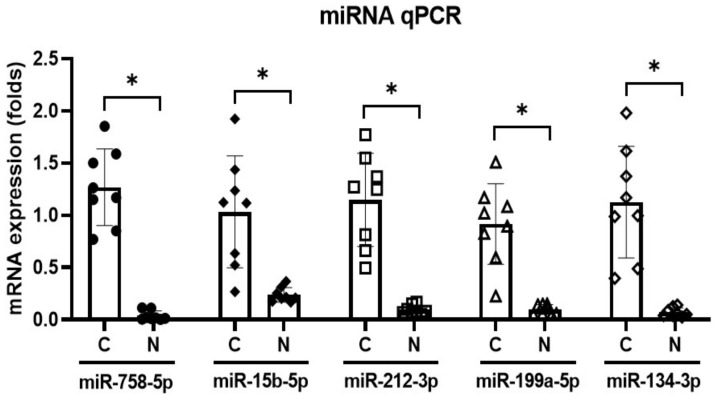
miR-758-5p, miR-15b-5p, miR-212-3p, miR-199a-5p, and miR-134-3p expression was reduced in the noise-exposed rats (*n* = 8 per group; * *p* < 0.05; unpaired *t*-test between control and noise-exposed rats).

The expression levels of miR-758-5p, miR-15b-5p, miR-212-3p, miR-199a-5p, and miR-134-3p were lower in the noise-exposed rats than in the control rats (1.27 [SD = 0.37] vs. 0.04 [SD = 0.05], *p* < 0.001 for miR-758-5p; 1.03 [SD = 0.54] vs. 0.24 [SD = 0.07], *p* < 0.001 for miR-15b-5p; 1.15 [SD = 0.45] vs. 0.10 [ 0.04], *p* < 0.001 for miR-212-3p; 0.92 [SD = 0.39] vs. 0.10 [SD = 0.05], *p* < 0.001 for miR-212-3p; 0.92 [SD = 0.39] vs. 0.10 [SD = 0.05], *p* < 0.001 for miR-199a-5p; 1.13 [SD = 0.54] vs. 0.07 [SD = 0.04], *p* < 0.001 for miR-134-3p) (Figure 4).

**Figure 5:**
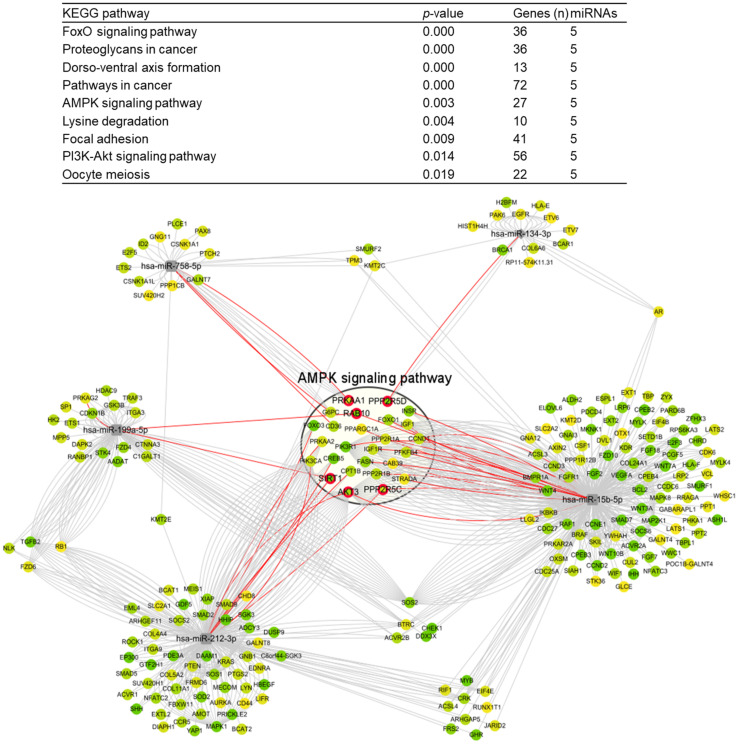
Pathways related to the dysregulated miRNAs in the cochlear nuclei of noise-exposed rats.

Pathways involving miR-758-5p, miR-15b-5p, miR-212-3p, miR-199a-5p, and miR-134-3p were analyzed (Figure 5). FoxO signaling, dorsoventral axis formation, AMPK signaling, lysine degradation, focal adhesion, and PI3K-Akt signaling were found to be associated with these downregulated miRNAs.

Among the predicted pathways, the AMPK signaling pathway was explored for related genes based on the previous studies which reported the modulation of AMPK signaling following noise-induced hearing loss [15,16]. Each miRNA was investigated for its associated target genes of the AMPK signaling pathway (Table 1). The predicted target genes of miR-758-5p–related AMPK signaling were *G6PC*, *PRKAA1*, and *SIRT1*. The predicted target genes of miR-15b-5p, i.e., *FASN*, *IGF1R*, *PPP2R5C*, *CCND1*, *PPP2R1A*, *PIK3R1*, *INSR*, and *IGF1*, were related to AMPK signaling. The predicted target genes of miR-212-3p, i.e., *PPP2R5C*, *AKT3*, *PIK3CA*, *FOXO3*, and *CPT1B*, were related to AMPK signaling. The predicted target genes of miR-199a-5p, i.e., *PRKAG2*, *RAB10*, *PRKAA2*, *SIRT1*, *CREB2*, and *PPP2R5C*, were involved in AMPK signaling. The predicted target gene of miR-134-3p was *PPP2R5D*.

**Figure 6:**
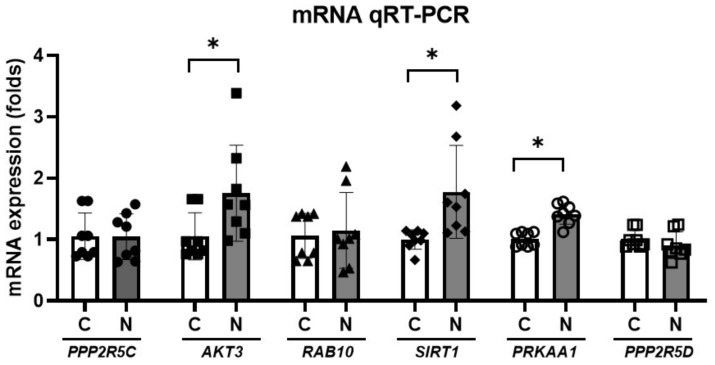
mRNA expression levels of *PPP2R5C*, *AKT3*, *RAB10*, *SIRT1*, *PRKAA1*, and *PPP2R5D* were higher in the noise-exposed rats than in the control rats (*n* = 8 per group; * *p* < 0.05; unpaired *t*-test between control and noise-exposed rats).

Among the predicted genes related to AMPK signaling, *PPP2R5C*, *AKT3*, *RAB10*, *SIRT1*, *PRKAA1*, and PPP2R5D were examined for their mRNA expression levels using qRT-PCR (Figure 6). The mRNA expression levels of *AKT3*, *SIRT1,* and *PRKAA1* were higher in the noise-exposed rats than in the control rats (1.05 [SD = 0.38] vs. 1.76 [SD = 0.78], *p* = 0.039 for *AKT3*; 1.00 [SD = 0.16] vs. 1.78 [SD = 0.76], *p* = 0.013 for *SIRT1*; 1.01 [SD = 0.11] vs. 1.42 [SD = 0.17], *p* < 0.001 for *PRKAA1*).

**Figure 7:**
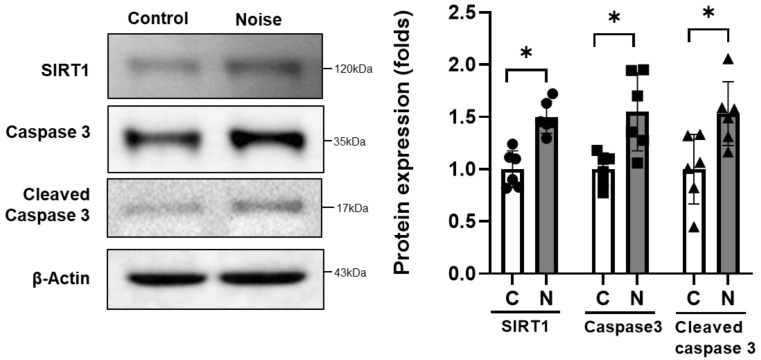
SIRT1, caspase 3, and cleaved caspase 3 expression in the cochlear nuclei after noise exposure (*n* = 6 per group). SIRT1, caspase 3, and cleaved caspase 3 expressions were higher in the noise-exposed group (* *p* < 0.05; unpaired *t*-test between control and noise-exposed rats; C: control rats; N: noise rats).

The protein expression level of SIRT1 was higher in the noise-exposed rats than control rats (1.00 [SD = 0.17] vs. 1.50 [SD = 0.15], *p* < 0.001, Figure 7). The protein expression levels related with apoptosis were also higher in the noise-exposed rats than control rats (1.00 [SD = 0.16] vs. 1.55 [SD = 0.37], *p* = 0.013 for caspase 3; 1.00 [SD = 0.33] vs. 1.53 [SD = 0.31], *p* = 0.016 for cleaved caspase 3).

## 3. Discussion

In the present study, the CN of noise-exposed rats demonstrated an increased expression of brevican. Five miRNAs, miR-758-5p, miR-15b-5p, miR-212-3p, miR-199a-5p, and miR-134-3p, were downregulated in the CN of noise-exposed rats. These downregulated miRNAs were related to the AMPK signaling pathway. Among the predicted target genes related to the AMPK signaling pathway, the mRNA expression of *AKT3* and *SIRT1* was increased in the CN of noise-exposed rats. These changes particularly in AMPK signaling pathway can be related with the increased expression of brevican.

In this study, several miRNAs were found to be differentially expressed in CN following chronic noise exposure. A number of prior studies described the plausible mechanisms of these miRNAs with neural plastic changes [17,18,19,20,21,22]. miR-15b was reported to control epigenetics by inhibiting the methylation of cyclin D1 through the downregulation of a key enzyme (methylcytosine dioxygenase) in the ten-eleven translocation family, thereby dysregulating the cell cycle and differentiation of neural progenitors and accelerating neurogenesis [17]. In patients with Alzheimer’s disease, blood levels of miR-15b were downregulated compared to those in the control participants [18]. miR-212 has been characterized as a regulator of synaptic plasticity following external stimuli [19]. The dysregulation of miR-212, both overexpression and conditional knockout, induces anxiety-like behaviors following acute and chronic stress exposure [19]. In the animal models with dysregulated miR-212 expression, *SIRT1* and *AKT* were suggested to be involved as target genes of miR-212 [19]. In addition, the expression levels of miR-212 were reduced in neutrally derived plasma exosomes from patients with Alzheimer’s disease [20]. miR-199a was suggested to inhibit neuritin in the hippocampus and cortex in the early stage of an Alzheimer’s disease model (APPSWE, PSEN1dE9 transgenic mice) [21]. miR-134 is mainly localized in the synapto-dendritic regions and is presumed to repress synaptic plasticity [10]. Following exposure to unpredictable chronic mild stress, miR-134 inhibits the phosphorylation of synapse-associated proteins of LIM-domain kinase 1 and cofilin, which reduces synaptic plasticity and induces depression-like behavior [22]. Thus, reduced miR-134 expression in the noise-exposed rats in the current study implied neural plastic changes induced by the noise stimulus.

The AMPK pathway involving SIRT1 and AKT was suggested as a target pathway for the downregulated miRNAs of CN in noise-exposed rats in the current study. AMPK and SIRT1 are evolutionarily similar proteins that share common target molecules to regulate metabolism and inflammation [23]. AMPK expression in the cochlear spiral ligament was transiently enhanced following noise exposure. AMPK is activated by pathological stresses such as hypoxia, oxidative stress, and noise exposure [16]. Upon the activation of AMPK, the expression of SIRT1 increases and induces signaling cascades involving AKT [24].

In the present study, SIRT1 expression was increased in the CN of noise-exposed rats. SIRT1 is a homolog of sirtuins (SIRT1–SIRT7), which are predominantly expressed in the central nervous system [25]. As an oxidized nicotinamide adenine dinucleotide-dependent histone and nonhistone deacetylase, SIRT1 responds to various external stimuli via deacetylation of several transcription factors associated with inflammation and apoptosis [26,27]. SIRT1 has been suggested to be crucial to determine stress-induced anhedonia, as it enhances anxiety and depression [28]. Although the pathophysiological mechanism is still elusive, activation of SIRT1 is supposed to reinforce signaling cascades, such as ERK1/2 cascades, and inhibit neurogenesis [28,29]. SIRT1 activates AKT expression and promotes axonogenesis [30]. In animal models exposed to external stimuli, such as cerebral ischemia and exercise, SIRT1 was upregulated with the activation of AKT signaling cascades and pro-apoptotic caspase 3 expression [24,31].

In this study, expression of *AKT3* was increased in the CN of noise-exposed rats. AKT was upregulated in selective nerve fibers of the osseous spiral lamina of the cochleae in noise-exposed guinea pigs [32]. AKT is activated by distress, including ischemia and noise exposure, and activates a number of survival pathways, including counteracting apoptosis [33,34]. As most cell types of the organ of Corti, stria vascularis, and supporting cells exhibited reduced expression of AKT following noise exposure, it was presumed that noise distress induced pro-apoptotic responses, and selective nerve fibers that showed increased AKT expression might be more responsive to noise stimuli, which led to early spatial activation of the antiapoptotic pathway [32]. Dysregulation of AKT signaling has been demonstrated in the cochleae of animals with noise-induced, aminoglycoside-induced, and aging-related hearing loss [35,36,37,38].

The expression of brevican is increased in the CN of noise-exposed rats of the present study. Although the molecular mechanisms underlying the changes in brevican expression in the CN are elusive, the elevated AKT levels could be associated with the increased expression of brevican in the CN of noise-exposed rats. Although the molecular mechanisms linking AMPK signaling involving SIRT1 and AKT with CSPGs is still elusive, a prior study suggested the AMPK signaling induced regulation of CSPGs in brain tumor [39]. In addition, It has been reported to induce the expression of neurocan and brevican in astrocytes [40]. High levels of CSPGs, such as Neurocan and Brevican, inhibit axonal growth by inducing astrogliosis and glial scarring [41,42]. Thus, the high levels of AKT might be related to the high expression of brevican in this study. In addition, increased expression of brevican may induce glial scarring and inhibition of axonal regeneration in the CN following chronic noise exposure. Although the auditory threshold shifts were examined, the quantitative analyses on the cochleae cannot be conducted in this study due to the small number of study group. Because the noise exposure condition of the current study was enough to induce permanent threshold shifts, we can presume the cochlear dysfunction and injuries through all turns of cochleae. Further studies are needed to delineate the specific miRNAs inducing molecular cascades involving AKT and brevican in the CN following chronic noise exposure. Moreover, the clinical relevance of these molecular pathways in chronic noise-induced hearing loss condition without total deafness should be unraveled in follow-up research.

## 4. Materials and Methods

### 4.1. Noise and Comparison Groups

The Institutional Animal Care and Use Committee of CHA University Medical School (IACUC190046) approved this study. All experimental procedures followed the guidelines of the Institutional Animal Care and Use Committee of CHA University Medical School. The rats (postnatal eight-week Sprague-Dawley) were divided into control and noise-exposed groups (Figure 1). The rats in the noise-exposed group were exposed to wideband noise (2–20 kHz, 115 dB SPL, 4 h/day for 3 days/week for 4 weeks) in free-field condition using a speaker placed at the ceiling of the noise box (Tucker-Davis Technologies; Alachua, FL, USA). The 115 dB SPL of noise was applied to result in a permanent threshold shift. We chose the profound hearing loss animals to exclude the neural plasticity due to the recovery of hearing loss, which can be mixed up with the neural plasticity from the auditory deprivation. Rats were not anesthetized during noise exposure and the noise box was soundproofed. Rats in the control group were raised with ambient noise of approximately 40–60 dB SPL. Hearing levels were estimated using auditory brainstem response (ABR) thresholds. Two months after noise exposure, all rats were euthanized and the cochleae and CN were dissected [43].

### 4.2. Hearing Level Measurement

Hearing levels were measured using SmartEP Intelligent Hearing System (Miami, FL, USA) [44]. ABR measurements were conducted under anesthesia (40 mg/kg zoletil and 10 mg/kg xylazine). The reference, ground, and measuring electrodes were placed in the vertex, contralateral thigh, and ipsilateral retroauricular space, respectively. A plastic earphone was placed into the ipsilateral ear canal and connected to an EC1 electrostatic speaker. Pure tone sound stimuli were delivered to earphones at frequencies of 4, 8, 16, and 32 kHz (duration: 1562 µs; envelope: Blackman; stimulation rate: 21.1/s; amplitude: 90–20 dB SPL, 1024 sweeps). The hearing threshold was set as the lowest sound amplitude-evoked wave II [45].

### 4.3. Protein Expression in CN

The expression levels of arc, neurocan, brevican, SIRT1, caspase 3, and cleaved caspase 3 were evaluated in the CN. The protein was purified from CN tissue using a lysis buffer (Pre-prep, Intron). Equal amounts of protein samples were subjected to 8–10% sodium dodecyl sulfate-polyacrylamide gel electrophoresis. After transferring the resolved proteins onto polyvinylidene difluoride membranes (Merck Millipore, Burlington, MA, USA), they were incubated with 1:1000 anti-arc (Santa Cruz, Dallas, TX, USA, sc166461), anti-neurocan (Mybiosource, San Diego, CA, USA, MBS822310), anti-brevican (Santa Cruz, Dallas, TX, USA, sc135849), anti-SIRT1 (Santa Cruz, Dallas, TX, USA, sc74465), caspase 3 (Cell Signaling Technology, Danvers, MA, USA, 9662s), cleaved caspase 3 (Cell Signaling Technology, 9661s), and anti-rabbit monoclonal β-actin antibodies (Cell Signaling Technology, D6A8). Horseradish peroxidase (HRP)-conjugated secondary antibodies were treated with 1:2000 anti-rabbit IgG, HRP-linked (Cell Signaling Technology, 7074s), and goat anti-mouse IgG H&L (HRP) (Abcam, Cambridge, England, ab97023). The membranes were visualized using an enhanced chemiluminescence kit (Bio-Rad, Hercules, CA, USA). Protein bands were quantified using the ImageJ software (National Institutes of Health, Bethesda, MD, USA).

### 4.4. miRNA Expression in the CN

The miRNAs that showed expression changes following noise exposure or hearing loss in the central nervous system were examined in the CN of rats exposed to chronic noise. CN from eight rats per group were analyzed. MDNA was synthesized from purified miRNAs using the miScript^®^ II RT kit (Qiagen, Hilden, Germany). PCR was conducted using the miScript SYBR^®^ Green PCR kit (Qiagen) with the miScript Primer Assay reagents (Qiagen). U6 small nuclear RNA was used as the reference. ABI 7500 real-time PCR system (Applied Biosystems, Foster City, CA, USA) was used. The expression levels of miRNAs were quantified using cycle threshold (Ct) values and the 2^−∆∆Ct^ method.

### 4.5. The Predicted Target Genes for miRNAs

The predicted target pathways and related genes for the dysregulated miRNAs were accessed using DIANA-mirPath v.3 (Available online: http://snf-515788.vm.okeanos.grnet.gr/ (accessed on 7 June 2021)). KEGG pathways and the related target genes were searched with a threshold of 0.8 and a false discovery rate correction [46].

The mRNA expression of the predicted target genes was analyzed using quantitative reverse-transcription-PCR (qRT-PCR) [47,48]. Following reverse transcription of cDNA from purified RNA, the reagent was prepared using TOPreal™ qPCR 2× PreMIX (SYBR Green with low ROX; Enzynomics; Daejeon, Korea). PCR was conducted using the ViiA7 real-time PCR system (Applied Biosystems, Carlsbad, CA, USA). The primer sets used in this study are listed in Table 2. Glyceraldehyde 3-phosphate dehydrogenase (*GAPDH*) was used as the reference gene. mRNA expression levels were quantified using the 2^−^^ΔΔCt^ method.

### 4.6. Statistical Analysis

The hearing threshold shifts after the noise exposure were compared between nose-exposed and control rats using a unpaired *t*-test. The mRNA and miRNA expression levels in noise-exposed and control rats were compared using the unpaired *t*-test. The results are presented as mean ± standard deviation (SD). All analyses were conducted using the SPSS software (ver. 21.0; IBM Corp.; Armonk, NY, USA). Statistical significance was set at *p* ≤ 0.05.

## 5. Conclusions

The CN of noise-exposed rats exhibited elevated expression of arc and brevican. The levels of several miRNAs, including miR-758-5p, miR-15b-5p, miR-212-3p, miR-199a-5p, and miR-134-3p, were low in the CN of rats exposed to chronic noise. The AMPK signaling pathway involving *AKT3* and *SIRT1* was predicted to be activated in noise-exposed rats.

## Figures and Tables

**Table 1:** The predicted target genes related to the AMPK signaling pathway of the downregulated miRNAs in the cochlear nuclei of noise-exposed rats.

Related miRNA	Gene Name		Score
hsa-miR-15b-5p	FASN	fatty acid synthase	0.935
	IGF1R	insulin like growth factor 1 receptor	0.866
	PPP2R5C	protein phosphatase 2 regulatory subunit B’gamma	0.845
	CCND1	cyclin D1	0.873
	PPP2R1A	protein phosphatase 2 scaffold subunit Aalpha	0.847
	PIK3R1	phosphoinositide-3-kinase regulatory subunit 1	0.989
	INSR	insulin receptor	0.947
	IGF1	insulin like growth factor 1	0.859
	AKT3	AKT serine/threonine kinase 3	1
	PPARGC1A	PPARG coactivator 1 alpha	0.806
	FOXO1	forkhead box O1	0.913
	CAB39	calcium binding protein 39	0.8
	PPP2R1B	protein phosphatase 2 scaffold subunit Abeta	0.885
	RAB10	RAB10, member RAS oncogene family	0.941
	PFKFB4	6-phosphofructo-2-kinase/fructose-2,6-biphosphatase 4	0.872
	STRADA	STE20 related adaptor alpha	0.81
hsa-miR-758-5p	CD36	CD36 molecule	0.901
	G6PC	glucose-6-phosphatase catalytic subunit 1	0.865
	PRKAA1	protein kinase AMP-activated catalytic subunit alpha 1	0.84
hsa-miR-199a-5p	SIRT1	sirtuin 1	0.9
	PRKAG2	protein kinase AMP-activated non-catalytic subunit gamma 2	0.831
	RAB10	RAB10, member RAS oncogene family	0.954
hsa-miR-212-3p|	PRKAA2	protein kinase AMP-activated catalytic subunit alpha 2	0.844
	SIRT1	sirtuin 1	1
	CREB5	cAMP responsive element binding protein 5	0.982
	PPP2R5C	protein phosphatase 2 regulatory subunit B’gamma	0.889
	AKT3	AKT serine/threonine kinase 3	0.92
	PIK3CA	phosphatidylinositol-4,5-bisphosphate 3-kinase catalytic subunit alpha	0.864
	FOXO3	forkhead box O3	0.997
	CPT1B	carnitine palmitoyltransferase 1B	0.892
hsa-miR-134-3p	PPP2R5D	protein phosphatase 2 regulatory subunit B’delta	0.965

**Table 2:** Oligonucleotide primer sequences for quantitative reverse transcriptase polymerase chain reaction.

Gene	Primer Sequence (Forward)	Primer Sequence (Reverse)	Annealing Temperature (°C)	Product Size (bp)
*PPP2R5C*	5′-CTAGCCAAAGCGAATCCCCA-3′	5′-GAGTCTCGTCGCTCACTGTC-3′	60	93
*AKT3*	5′-CCACCTGAAAAGTATGACGACG-3′	5′-TAAGAGCGAGGACTGGTGGA-3′	60	133
*RAB10*	5′-TTGTTTGCCCCCACTACTCC-3′	5′-TAAATGAGGGGCTGACACCG-3′	60	185
*SIRT1*	5′-GCAGGTTGCGGGAATCCAA-3′	5′-GGCAAGATGCTGTTGCAAA-3′	60	155
*PRKAA1*	5′-GGGTGAAGATCGGCCACTAC-3′	5′-CTCTCTGCGGATTTTCCCGA-3′	60	62
*PPP2R5D*	5′-CGAGTCGGGTCGCTAAGAAG-3′	5′-ACACTCAGAGTCAAAGGGCG-3′	60	164
*GAPDH*	5′-AGTGCCAGCCTCGTCTCATA-3′	5′-AAGAGAAGGCAGCCCTGGTA-3′	60	93

## Data Availability

The data presented in this study are available upon request from the corresponding author.
